# Quality of Low-Allergy Wheat (‘O-Free’) Flour and Optimization of Its Bread-Baking Performance

**DOI:** 10.3390/foods11213399

**Published:** 2022-10-27

**Authors:** Tianyi Xia, Kyeonghoon Kim, Meera Kweon

**Affiliations:** 1 Department of Food Science and Nutrition, Pusan National University, Busan 46241, Korea; 2 Wheat Team, National Institute of Crop Science, Rural Development Administration, Jeonju 55365, Korea; 3 Kimchi Research Institute, Pusan National University, Busan 46241, Korea

**Keywords:** low-allergy wheat, flour quality, optimization, bread-baking performance, response surface methodology

## Abstract

This study explored the quality of hypoallergenic wheat (’O-free’) developed in Korea and optimized the basic ingredients and processing conditions for making ‘O-free’ bread using response surface methodology. Water and yeast amounts and mixing and fermentation times were selected as factors, and each factor’s tested range was set by a central composite design using Design Experts: water 52–60 g, yeast 1.5–4.5 g, mixing time 2.5–5 min, and fermentation time 50–70 min. Bread height, volume, and firmness were analyzed to determine bread quality. Flour quality analysis showed that ‘O-free’ flour’s gluten strength was weak. ‘O-free’ flour exhibited inferior bread-making performance compared to representative bread flour. Water and yeast amounts and mixing time, except for fermentation time, affected bread quality significantly. The interaction between yeast and fermentation also affected bread quality significantly. The optimized condition for making bread using ‘O-free’ flour is 60 g of water, 2.6 g of yeast, 2.5 min of mixing time, and 70.0 min of fermentation time. In conclusion, ‘O-free’ flour with the changed gluten composition showed poor gluten strength and bread-making performance. However, modifying the formulation of the basic ingredients and processing conditions could significantly improve the production of high-quality hypoallergenic bread.

## 1. Introduction

Wheat is a highly adaptable and widely distributed cereal grain with high nutritional value globally, providing approximately 21% of food calories and 20% of the protein people need. Approximately 35–40% of the world’s population consumes wheat as their staple food. Wheat consumption in Korea is approximately 2 million tons per year and the per capita annual consumption of 34.2 kg [1]. However, the domestic wheat self-sufficiency rate is currently less than 1%, and most of the wheat used in confectionery, noodles, and bread in Korea is imported from countries such as the United States, Australia, and Canada [1]. Efforts to promote the production and consumption of domestic wheat have been continuously made in breeding, cultivation, and processing. To secure competitiveness and differentiate the quality of Korean domestic wheat, the National Institute of Crop Science in Korea has developed an ‘O-free’ wheat variety that is free of ɷ-5 gliadin (a major wheat allergen) and is reduced low molecular weight (LMW) glutenin by missing Glu-B3 trait, γ-gliadin, and α-amylase inhibitor. The absence of ɷ-5 gliadin was further analyzed/confirmed by acid polyacrylamide gel electrophoresis (A-PAGE) and expressed sequence tags (ESTs) [2,3,4]. As a representative wheat product, bread has gradually increased its consumer market along with westernizing living and eating habits. Therefore, producing low-allergy bread using ‘O-free’ flour can boost high-added value. 

Approximately 15% and 80–85% of wheat is composed of protein and gluten protein, respectively [5]. Gluten protein, which exists in gliadin and glutenin, is an important factor contributing to the quality of wheat flour and products. However, in people suffering from a wheat allergy, which has an antigen-antibody reaction to gluten, it can become an allergen and cause symptoms such as diarrhea, bloating, weight loss, anemia, and skin rash [6,7]. Compared with nearly 5% of wheat allergies in Western countries, wheat allergies are not common among Asians; however, the existence of this group cannot be ignored. In addition, consumers’ complaints such as bloating increase when eating wheat-based foods in Korea, although the severity of the wheat allergy is weaker than celiac disease [8]. Therefore, it is necessary to pay attention to allergens in food to reduce or eliminate the food’s allergenicity. Most staple foods, such as bread, noodles, and snacks, are cereal products that contain gluten [9]. Patients with celiac disease need to avoid foods containing gluten. Recent progress has been made in developing gluten-free products such as bread made with various gluten-free grains crops such as rice, corn, potato, tapioca, oat, and buckwheat as well as starch materials. Mainly, for replacing the gluten network in developing gluten-free bread, a wide range of functional additives have been investigated: proteins, hydrocolloids, fibers, enzymes, and emulsifiers. In addition, technological approaches such as sourdough and non-conventional technologies like high hydrostatic pressure and ohmic heating reported promising results [10,11,12]. However, substituting gluten in cereal products remains a major technical challenge [13].

Gluten is an essential structural building protein widely used to fortify low-protein flour to produce excellent baked goods. It forms a gluten network structure through interchain and intermolecular disulfide bonds, providing dough with viscoelasticity, good gas retention, and good crumb structure for the resulting baked products [13,14,15]. The interaction between gluten and starch significantly impacts the quality of dough and its products [16]. Many studies have shown that a lack of gluten reduces bubbles entrained in the dough matrix and prevents increased volume required during fermentation and baking [17]. Gliadins are comprised of α- and γ-gliadin as sulfur-rich prolamin (6 & 8 cysteine residues, respectively) and ɷ-gliadin as sulfur-poor prolamin (0 cysteine residue). Glutenins comprise LMW and high molecular weight (HMW) subunits [18]. For developing gluten, the number of cysteine residues in each gliadin and glutenin component is important due to the availability for forming intra- and inter-disulfide (SS) bonds. Gliadins, except for ɷ-gliadin, and LMW and HMW glutenin subunits contribute to SS bonds [5]. Therefore, bread made from ‘O-free’ flour with decreased LMW glutenin subunits and γ-gliadin could be expected to have inferior quality compared to bread made from regular wheat. Therefore, an improvement in the quality of bread made with ‘O-free’ flour is needed to meet consumer preferences. The general method to improve the quality of bakery products is to change the basic ingredient formulations and processing conditions or to add vital wheat gluten as a dough improver [19]. However, the production of bread made with ‘O-free’ flour with vital wheat gluten is undesirable due to increased allergenicity. Therefore, it is useful to apply the former approach for improving bread quality of ‘O-free’ flour.

This study explored the effect of changes in the gluten composition of low-allergy wheat ‘O-free’ flour on flour quality and bread-making performance. Commercial bread flour and the ‘Baekkang’ (Korean domestic wheat cultivar for bread making) flour were used as the control group and compared with ‘O-free’ flour. Using response surface methodology (RSM), the basic ingredients and processing conditions were optimized for producing high-quality bread. Four factors were selected based on the central composite design for optimization: water amount and yeast amount as ingredients, mixing and fermentation time as processing conditions. Each factor was tested at two levels, including the center points. Bread quality was analyzed based on bread height, volume, and texture.

## 2. Materials and Methods

### 2.1. Materials

The ‘O-free’ (low-allergy wheat) flour and ‘Baekkang’ flour (designated as bread flour A: BF-A) were supplied from the National Institute of Crop Science in Korea, and a commercial bread flour (designated as bread flour B: BF-B) (Q1, Samyang, Seoul, Korea) was purchased at a local market. The protein content of the ‘O-free’ flour, BF-A, and BF-B was 11.5, 13.2, and 14.0% (14% mb), respectively. Ingredients for baking bread were also purchased from a local market. All chemicals were reagent grade for testing the solvent retention capacity (SRC) and sodium dodecyl sulfate (SDS) sedimentation volume. 

### 2.2. Evaluation of Flour Quality Characteristics

#### 2.2.1. SRC Analysis of Flour

The SRC analysis was conducted according to the Method 56-11.02 [20] to evaluate flour quality. Distilled water, 5% (*w*/*w*) sodium carbonate, 5% (*w*/*w*) lactic acid, and 50% (*w*/*w*) sucrose were used as the solvents. The flour suspension (5 g flour and 25 g of each solution) in a conical tube was dispersed and hydrated for 20 min and centrifuged at 1000× *g* for 15 min (LaboGene1248, Gyrozen Inc., Daejeon, Korea). After removing the supernatant, the tube was inverted for 10 min. The SRC was calculated based on the weight of the tube with the pellet.

#### 2.2.2. Measurement of SDS-Sedimentation Volume of Flours

The SDS sedimentation volume of the flour samples was measured using the method described by Axford et al. [21]. The flour suspension (5 g flour and 50 mL of distilled water) in a 100 mL graduated cylinder with a lid was shaken vigorously horizontally for 15 s and inverted approximately 10 times within 15 s at 2, 4, and 6 min to dissolve the flour sample completely. The cylinder was kept parallel to the desktop without shaking. After adding 50 mL SDS-lactic acid (3% SDS in 1.2N lactic acid), the cylinder was inverted 10 times in 15 s at 0, 2, 4, and 6 min in the previous steps. The cylinder was upright, and the cylinder scale was recorded at 20/40/60 min as the sedimentation volume for judging gluten quality.

#### 2.2.3. Measurement of Dough Mixing Property

The dough characteristics of flour were evaluated using Method 54-40.02 [20]. A flour sample (10 g) was placed in a mixing bowl, and distilled water (5.6–7.0 g), based on the water absorption values of flour by SRC, was added to the bowl containing the flour sample. The flour and water were mixed for 10 min using a Mixograph (10 g Mixograph, National Manufacturing Co., Lincoln, NE, USA), and the mixogram was recorded. 

### 2.3. Bread Making and Quality Evaluation

#### 2.3.1. Preparation of Bread

The bread was prepared using a slightly modified Method 10-10.03 [20]. As shown in Table 1, flour, non-fat dry milk, salt, and shortening were weighed and placed in the mixing bowl of a pin mixer (100 g, National Manufacturing Co., Lincoln, NE, USA). Sugar and yeast were separately added to distilled water (56 g for ‘O-free flour, 69 g for Bread flour A & B) and stirred until they were completely dissolved. The sugar-yeast mixed solution was poured into the mixing bowl and mixed for 3 min for ‘O-free flour and 5 min for Bread flour A & B. The prepared dough was pressed to 0.47 cm thickness using a dough sheeter (YT-160, Shanghai Huayuan Food Machinery Co., Ltd., Shanghai, China), folded, and placed in a baking pan (7 × 15 × 6.5 cm). The dough was fermented in a fermentation chamber (Phantom M301 Combi, Samjung, Gyeonggi, Korea) at 35 °C and 85% RH for 60 min.

The fermented dough was baked at 215 °C for 18 min in an oven (Phantom M301 Combi; Samjung, Gyeonggi, Korea). The baked bread was cooled and removed from the pan. Moisture loss during baking was calculated based on the recorded dough and bread weights. In addition, dough height before and after fermentation and bread height were measured.

#### 2.3.2. Evaluation of Bread Quality Characteristics

Dough heights before and after fermentation and bread height were measured using a caliper (HDS-20C, Mitutoyo, Kanagawa, Japan). The degree of expansion of the dough was calculated from the change in dough height. The bread volume was measured according to a slight modification of Method 10-05.01 [20]. This study used glutinous millet as a seed to fill a container (1.5 L). The bread was placed in a container, and overflowed glutinous millet was weighed. According to the specific volume of glutinous millet (mL/g), the weight was converted into volume to obtain the volume of bread (mL). Each bread was measured at least twice, and the average value was calculated. The bread firmness was measured force in compression with a probe (TA AACC36) on a 1.5 cm thick bread slice using a texture analyzer (CT3, Brookfield, Middleboro, MA, USA) according to Method 74-09.01 [20] at the following conditions: 2.0 mm/sec pretest speed; 2.0 mm/sec test speed; 5.0 mm/sec post-test speed; 10 mm penetration distance.

### 2.4. Design for Optimizing Formula and Processing Conditions of Bread

The Design Expert 10 (Stat-Easy Co., Minneapolis, MN, USA) program was used for the experimental plan based on a central composite design using response surface methodology. Based on a preliminary study using a factorial design, water, yeast, mixing time, and fermentation time were selected as factors. It is also well known that water and mixing time are essential for developing gluten, and yeast and fermentation time are important for leavening bread dough. Table 2 shows the four factors and the levels (− and +) for each factor. The center point (level 0) was water 56 g of water, 3.0 g of yeast, 3.0 min mixing time, and 60 min fermentation time. All experimental points are listed in the quality parameters measured. Bread height, volume, and firmness were selected as major quality responses. 

### 2.5. Statistical Analysis

Analysis of variance (ANOVA) and Tukey’s honestly significant difference (HSD) test (SPSS ver. 25.0, IBM Corp., Armonk, NY, USA) were used to analyze data and average comparisons between samples at *p* < 0.05. The Design Expert program was used to analyze the data from the experiments designed using RSM. According to the analysis of variance, adequate models were developed by identifying significant contributing factors and fitting response surface reduced quadratic models, and 3D plots were obtained. The optimum conditions for the amount of water and yeast and mixing and fermentation time were determined by setting the maximum bread volume and height and minimum bread firmness.

## 3. Results and Discussion

### 3.1. SRC of the Flours

**Figure 1:**
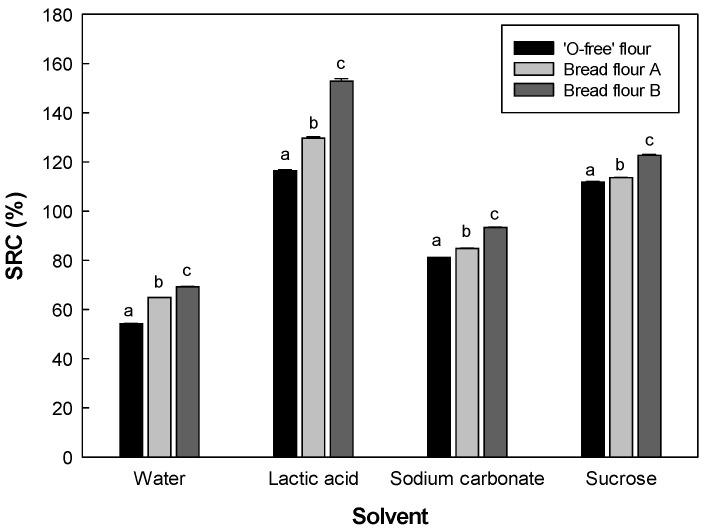
Solvent retention capacity of the flour samples: ‘O-free’ flour, Bread flour A, and Bread flour B. The different letters above the bars are significantly different at *p* < 0.05, according to Tukey’s HSD test.

The SRC results of the flour samples are shown in Figure 1. ‘O-free’ flour showed significantly lower SRC values in all four solvents than the bread flours, BF-A, and BF-B. Water SRC reflects the water absorption of flour, which is related to the amount of water required for mixing and forming a machinable dough [22]. Water SRC results indicate that ‘O-free’ flour requires less water to form the dough than BF-A and BF-B. The sodium carbonate SRC reflects the impact of damaged starch on flour characteristics [22]. The SRC value of ‘O-free’ flour in sodium carbonate solution was lower than those of BF-A and BF-B, which indicated the low contribution of damaged starch in ‘O-free’ flour. Lactic acid SRC values can be effectively used to determine the characteristics of gluten in the flour to determine the quality of the flour protein. In addition, lactic acid SRC is significantly correlated with protein content [23]. The protein content of the flours used in the study was in agreement with the correlation. Xiao et al. [24] and Boehm et al. [25] reported a high correlation between lactic acid SRC and bread volume, confirming the correlation between protein quality and final product quality. In this study, the lactic acid SRC value of ‘O-free’ flour was significantly lower than those of BF-A and BF-B. Therefore, the gluten strength of ‘O-free’ flour with the reduced low molecular glutenins was weak, which would be ineffective in producing gluten during the mixing process, affecting the dough property and negatively affecting the baking performance. It can be predicted that bread made from ‘O-free’ flour with relatively low lactic acid SRC value will be firmer and smaller than those made from BF-A and BF-B. The sucrose solution (50% *w*/*w*) exhibited good solvent compatibility with the xylan backbone of wheat flour arabinoxylans, and the swelling of the arabinoxylan network can be exaggerated in the solution, which can predict the arabinoxylan contribution of the flour [22,26]. Ram et al. [27] demonstrated that damaged starch and arabinoxylans control water absorption. The water absorption capacity of flour increases with an increase in damaged starch and arabinoxylans. The sucrose SRC value of ‘O-free’ flour was significantly lower than those of BF-A and BF-B, indicating a lower contribution of arabinoxylan and water absorption of ‘O-free’ flour.

### 3.2. SDS-Sedimentation Volume of Flours

**Figure 2:**
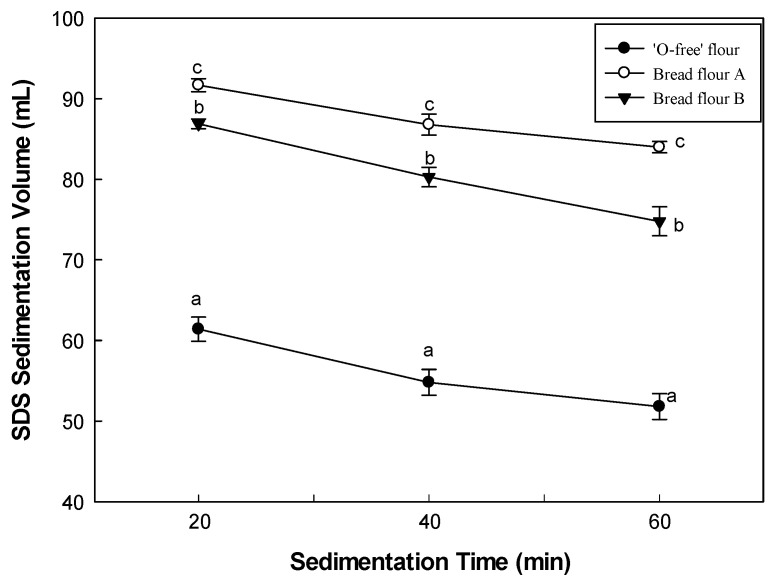
SDS sedimentation volume of the flour samples. The different letters above the symbols in line plots are significantly different at *p* < 0.05, according to Tukey’s HSD test.

The SDS sedimentation volume is based on the swelling of gluten in the SDS/lactic acid solution. It can be used to obtain a semi-quantitative estimate of the gluten content [28]. The SDS-sedimentation volumes of the flour samples are presented in Figure 2. The SDS-sedimentation volume of ‘O-free’ flour measured at 20 min of sedimentation time was 61.4 mL, and those of BF-A and BF-B were 91.7 and 86.88 mL, respectively. The result indicated that the gluten strength of ‘O-free’ flour was relatively weak due to the absence in parts of low molecular glutenins. The sedimentation volume of the ‘O-free’ flour, BF-A, and BF-B at 40 and 60 min of sedimentation time are 54.8, 86.8, and 80.3 mL, and 51.8, 84.7, and 75.0 mL, respectively. The sedimentation volume of ‘O-free’ flour was significantly lower than those of BF-A and BF-B at all sedimentation times, and all flours gradually decreased with increasing sedimentation time due to settling by gravity. In Figure 2, the slope of ‘O-free’ flour seemed to be relatively large, which reflected the weaker gluten strength of ‘O-free’ flour compared to BF-A and BF-B. SDS sedimentation volume is closely related to protein content, gluten index, wet gluten content, farinograph parameters, and bread quality. Bread volume and bread crumb structure were positively affected by the SDS sedimentation volume. Wheat flour with a higher SDS sedimentation volume tends to perform well in bread baking [24,29,30]. Therefore, it can be predicted that the bread made of ‘O-free’ flour with a low SDS-sedimentation volume might have a smaller bread volume and a poor bread crumb structure. 

### 3.3. Dough Mixing Property of Flours

**Figure 3:**
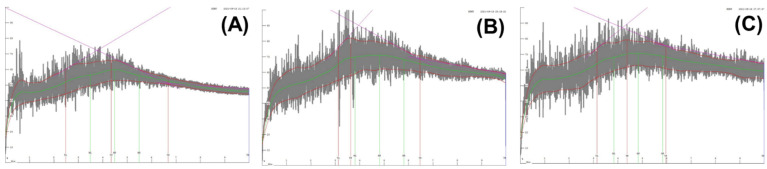
Mixograms of the flour samples: (**A**), ‘O-free’ flour; (**B**), Bread flour A; (**C**), Bread flour B.

Figure 3 shows the dough mixing patterns (mixograms) of the flour samples, providing information on mixing time requirement and tolerance. The mixing pattern of ‘O-free’ flour differed significantly from those of BF-A and BF-B. Although the time to reach the peak of ‘O-free’ flour was similar to those of BF-A and BF-B, the midline peak height of ‘O-free’ flour was lower, and the bandwidths of the peak and after reaching the peak were much narrower than those of BF-A and BF-B, indicating poor mixing tolerance. Moonen et al. [31] reported that the peak height of the mixogram is related to the flour’s protein content. Therefore, it could be considered that the protein content and dough viscoelasticity of ‘O-free’ flour were lower than those of BF-A and BF-B, which negatively impacted bread quality. Additionally, the reduced low molecular weight glutenin in ‘O-free’ flour might contribute to decreased glutenin macropolymer content and gluten development. Gil-Humanes et al. [32] reported the effect of low molecular weight (LMW) glutenin on mixing quality using transgenic lines, and low-LMW lines showed weaker mixing stability and tolerance than high-LMW lines. 

### 3.4. Quality Characteristics of Bread Prepared with Flours

**Figure 4:**
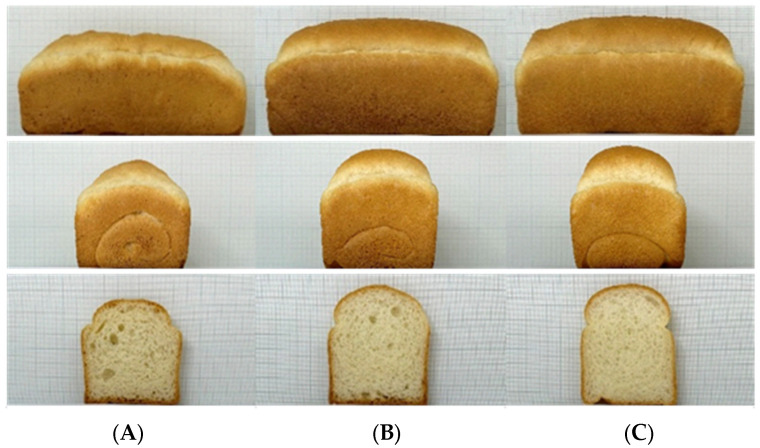
Side and cross-section views of bread made with the flour samples: (**A**), ‘O-free’ flour; (**B**), Bread flour A; (**C**), Bread flour B.

Figure 4 shows the photos of bread prepared with ‘O-free’ flour. The height and volume of the bread made of ‘O-free’ flour were significantly smaller than those of BF-A and BF-B. A strong correlation has been reported between protein content and bread volume of wheat flour: bread volume increases with the increased protein content of the flour, and the gluten index parameter is significantly related to the bread height to diameter [24,25,33,34]. In addition to the lower protein content of ‘O-free’ flour than those of BF-A and BF-B, the gluten strength of the dough with ‘O-free’ flour was weak, resulting in the dough being unable to retain gas during the fermentation and baking process, and the bread led to reduced volume and height. The change in dough height after fermentation to bread height reflected the effect of flour gluten strength. 

Table 3 shows the quality parameters of the bread made of the ‘O-free’ flour, BF-A, and BF-B. During baking, the moisture loss of bread made of ‘O-free’ flour was 8.7%, significantly lower than bread made of BF-A and BF-B (9.9 and 10.2%) because less water was added initially for preparing ‘O-free’ bread dough. Excessive moisture evaporation may cause the bread crust to thicken and the bread crumb to age faster [35]. Therefore, it is important to control moisture loss, which could make the bread expand properly, retain gas, and provide a desirable crumb texture. The firmness of bread made from ‘O-free’ flour was 7.8 N, significantly higher than that of BF-A and BF-B (4.8 and 3.7 N). Bread firmness may be related to the water absorption of flour [36]. Jo et al. [37] reported that when making dough, more water results in softer bread, which is similar to the results of this study.

Consumer expectations of products are generally determined through sensory evaluations [38]. The soft and elastic properties of breadcrumbs are usually considered favorable characteristics of bread quality [39,40]. Therefore, the bread made of ‘O-free’ flour showed small volume and height and large firmness, which would be undesirable and unfavorable attributes for the consumers in the traditional sense. The results suggested a need to improve the bread quality of ‘O-free’ flour to produce bread with reduced allergens. Optimizing formulation and processing conditions would be a promising approach because of the lack of supplemental additives and costs. 

### 3.5. Quality Analysis of Bread Made with ‘O-Free’ Flour Based on Response Surface Methodology

**Figure 5:**
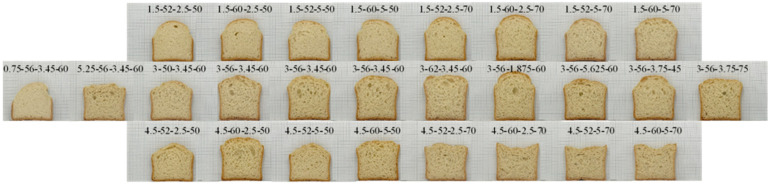
Cross-section views of bread prepared with ‘O-free flour’ at different formulation and processing conditions (yeast-water-mixing time-fermentation time) based on response surface methodology.

Figure 5 shows the cross-section of ‘O-free’ bread prepared under the test conditions. As the amount of water increased, the bread volume increased. However, when the yeast amount was higher than the center point, bread height decreased as the water amount increased. 

The fermented dough height and bread height are shown in Table 4. The fermented dough height was 36.6–76.4 mm, and the bread height was 52.3–74.5 mm. The bread height after baking increased or decreased depending on the amount of yeast: an increase with a lower amount of yeast (less than 1.5 g) and a decrease with a higher amount of yeast (more than 3.0 g). The height of the bread prepared at the center point was relatively high. The bread height was the highest for the bread made at 56.0 g of water, 3.0 g of yeast, 1.875 min of mixing time, and 60 min of fermentation time.

The volume of bread made with ‘O-free’ flour is shown in Table 4. The average bread volume was 343.8–536.2 mL. The largest bread volume was obtained with the conditions at 62.0 g of water, 3.0 g of yeast, 3.75 min of mixing time, and 60 min of fermentation time.

The firmness of bread made with ‘O-free’ flour was 3.1–17.1 N (Table 4). The bread prepared at 56.0 g water, 3.0 g yeast, mixing of 1.875 min, and fermentation of 60 min produced the lowest firmness near the center point. In addition, bread made under these conditions had the highest height and volume.

### 3.6. Optimizing Formula and Processing Conditions of Bread Made with ‘O-Free’ Flour

Among the checked adequate models, the Design Expert program suggested the quadratic model when fitting the data for all responses. However, the lack of fit for the responses except for bread volume was significant, suggesting improving adequacy by developing a reduced quadratic model. The ANOVA results for the reduced quadratic models are listed in Table 5. The models for all responses showed satisfactory coefficient (R^2^) ranging from 0.807 to 0.963 and insignificant lack of fit except for bread firmness due to a somewhat larger standard deviation of data than other responses. However, bread firmness is an important bread quality parameter in the industrial aspect and was selected for optimizing ingredient and processing conditions in the study.

Based on the developed models, significant model terms (*p* < 0.05) for each response were identified. Fermented dough height was significantly affected by all four factors and the interactions between water and yeast and yeast and fermentation time. Bread height was significantly affected by water, yeast, mixing time, and the interaction between yeast and fermentation time. Bread volume was significantly affected by water and mixing time. 

As dough ferments, yeast decomposes simple sugars and generates carbon dioxide and ethanol, which increases dough volume. As the bread dough is baked in the oven, more carbon dioxide is released, and the ethanol evaporates and turns into bubbles, causing the bread to expand. If the flour lacks gluten, air bubbles in the bread dough are lost, resulting in denser crumbs [41]. 

An adequate amount of water can improve the viscoelasticity of the dough and help the gluten network become firmer so that the bread expands to a larger volume. However, insufficient or excessive water affects the interaction between the ingredients in the dough [42]. ‘O-free’ flour lacks omega-gliadin in gluten protein. Even if a large amount of yeast is added to make the bread dough, the gluten network is fragile and easy to break at high temperatures, which causes a lower bread height than fermented dough. Furthermore, when an excess amount of water is added, the ‘O-free’ flour with a low water absorption rate cannot develop a strong gluten network, which causes a lower expansion than that of regular bread wheat flour. Eventually, the top surface of the bread becomes uneven and sags downward.

Water and the interaction between yeast and fermentation time significantly influenced bread firmness. Several studies have shown that soft bread had high moisture content and volume [20,37,43], similar to our results. The conditions for producing bread with the highest firmness were 56 g of water, 0.75 g of yeast, 3.75 min of mixing time, and 60 min of fermentation time. It is speculated that too little yeast prevents the bread from fully fermenting to form a gluten network to accommodate bubbles, and the relatively long mixing time leads to weaker dough elasticity.

**Figure 6:**
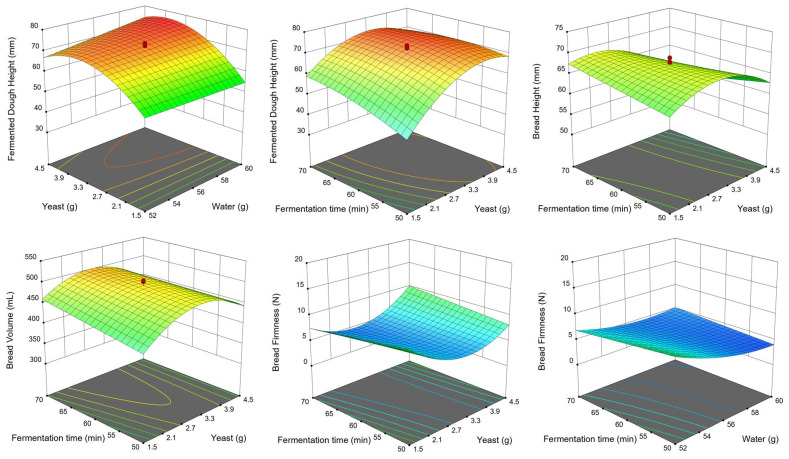
Response surface plots based on the reduced quadratic model developed for the fermented dough height, height, volume, and firmness of bread prepared with ‘O-free’ flour.

A three-dimensional diagram of each bread quality parameter is shown in Figure 6. Yeast and fermentation time reflected the most significant relationship with all measured responses. Bread height and volume increased, and firmness decreased as the yeast, and fermentation time approached the center point. The largest bread volume was obtained when the fermentation time was close to the center point.

**Figure 7:**
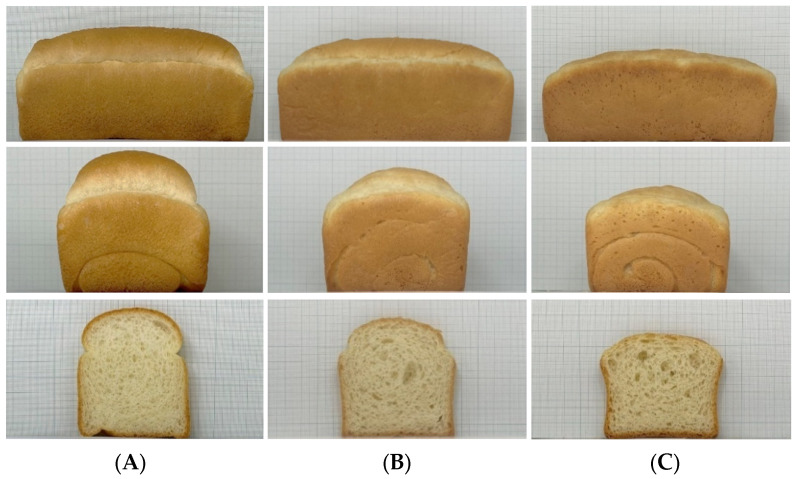
Side views and cross-sections of bread prepared with ‘O-free’ flour at the optimized conditions. (**A**), Bread with Bread flour B; (**B**) Bread with O-free flour prepared at the optimized condition; (**C**) Bread with O-free prepared at the center point.

Among the four factors set up in the experiment, fermentation time itself had no significant effect on the representative attributes of bread quality, such as bread volume, height, and firmness. However, the interaction between yeast and fermentation affected bread quality significantly. Furthermore, the water, yeast, and mixing time appeared to affect the quality. As the major quality parameter of bread, the maximum value of bread height and volume and the minimum value of bread firmness were set to identify the optimum conditions based on desirability. The optimized conditions were as follows: 60 g of water, 2.6 g of yeast, 2.5 min mixing time, and 70 min fermentation time. To validate the predicted value, one of the optimized solutions suggested by the Design Expert program was used to prepare bread and compare it with bread made at the center point (Table 6). The bread made with ‘O-free’ flour had a higher height, a larger volume, and a greater firmness than the predicted values. In addition, a significant improvement was observed compared to bread prepared with a center point (Figure 7). Overall, the quality of bread made with ‘O-free’ flour was significantly improved by optimizing formula and processing conditions.

## 4. Conclusions

As an allergy-reducing wheat variety, ‘O-free,’ developed by traditional breeding at the National Institute of Crop Science in Korea, lacks omega-gliadin. The effect of ‘O-free’ wheat flour on the qualitative characteristics of gluten protein and bread-making performance was explored. In addition, to improve the bread-making performance of ‘O-free’ flour, the basic ingredient formula and processing conditions were optimized using a response surface methodology. The lactic acid SRC value and SDS sedimentation volume of ‘O-free’ flour were significantly lower than those of the control bread flour samples, suggesting weaker gluten strength. The mixograms also showed a weak dough strength of ‘O-free’ flour as the bandwidth was reduced significantly after the mixing peak of the dough made from ‘O-free’ flour. Bread made with ‘O-free’ flour had significantly smaller height and volume and greater firmness than bread made with control wheat flour samples. The results suggested that the bread-making performance of ‘O-free’ flour was not excellent. The reduced quadratic model for each response was adequate and significant for fitting data. The significant model terms were the amount of water, yeast, mixing time, and interaction between yeast and fermentation. The optimized ingredient formula and process conditions were 60 g of water, 2.6 g of yeast, 2.5 min of mixing time, and 70.0 min of fermentation time, significantly improving the bread-making performance of ‘O-free’ flour. In conclusion, although the bread-making performance of ‘O-free’ flour itself was inferior, it could be significantly improved by optimizing the basic ingredient formula and process conditions. 

## Figures and Tables

**Table 1:** Ingredients and formula for bread prepared with various flours by Method 10-10.03 with a minor modification.

Ingredient	‘O-Free’ Flour	Bread Flour A	Bread Flour B
Flour (g)	100	100	100
Non-fat dry milk (g)	4	4	4
Salt (g)	1.5	1.5	1.5
Shortening (g)	3	3	3
Sucrose (g)	6	6	6
Yeast (g)	2	2	2
Water (g)	56	69	69

**Table 2:** Factors and levels of each factor in an experimental design based on response surface methodology.

Factor	Level		
	(−)	0	(+)
Water (g)	52	56	60
Yeast (g)	1.5	3	4.5
Mixing time (min)	2.5	3.75	5
Fermentation time (min)	50	60	70

**Table 3:** Quality characteristics of the bread prepared with the flour samples.

Quality Parameter		‘O-Free’ Flour	Bread Flour A	Bread Flour B
Moisture loss (%)		8.7 ± 0.4 ^a,(1)^	9.9 ± 0.3 ^b^	10.2 ± 0.3 ^b^
Dough Height (mm)	BF ^(2)^	37.7 ± 1.4 ^a^	36.5 ± 1.9 ^a^	36.7 ± 1.6 ^a^
	AF	66.5 ± 0.3 ^a^	69.2 ± 2.4 ^a^	72.9 ± 3.1 ^b^
Bread Height (mm)		73.6 ± 0.3 ^a^	79.5 ± 2.9 ^b^	83.2 ± 1.8 ^c^
Bread Volume (mL)		507.7 ± 11.8 ^a^	571.9 ± 21.2 ^b^	565.8 ± 9.3 ^b^
Bread Firmness (N)		7.8 ± 1.3 ^b^	4.8 ± 0.5 ^a^	3.7 ± 0.8 ^a^

^(1)^ Results are expressed as the mean ± SD. Values with the same letter within the same row are not significantly different (*p* < 0.05), according to Tukey’s HSD test. ^(2)^ BF, before fermentation; AF, after fermentation.

**Table 4:** Fermented dough height and height, volume, and firmness of bread prepared with ‘O-free’ flour.

Sample	Water (g)	Yeast (g)	Mixing Time (min)	Fermentation Time (min)	Responses			
					Fermented Dough Height (mm)	Bread Height (mm)	Bread Volume (mL)	Bread Firmness (N)
1	52	1.5	2.5	50	51.0 ± 0.1	63.9 ± 0.2	402.7 ± 1.0	12.1 ± 2.0
2	60	1.5	2.5	50	47.4 ± 0.1	64.9 ± 0.3	453.1 ± 3.1	9.0 ± 0.6
3	52	4.5	2.5	50	63.4 ± 0.4	59.3 ± 0.6	401.5 ± 2.1	14.7 ± 1.3
4	60	4.5	2.5	50	73.5 ± 0.4	67.1 ± 0.2	497.0 ± 1.1	4.9 ± 0.8
5	52	1.5	5	50	48.0 ± 0.1	62.9 ± 0.1	400.9 ± 0.5	12.5 ± 1.9
6	60	1.5	5	50	44.7 ± 0.3	63.1 ± 0.1	419.0 ± 0.3	12.4 ± 1.2
7	52	4.5	5	50	62.3 ± 0.3	58.3 ± 0.1	374.4 ± 0.2	14.3 ± 1.0
8	60	4.5	5	50	72.2 ± 0.3	63.2 ± 0.6	453.4 ± 3.4	7.7 ± 0.9
9	52	1.5	2.5	70	60.4 ± 0.3	67.5 ± 0.2	448.5 ± 0.3	7.4 ± 0.9
10	60	1.5	2.5	70	61.2 ± 0.2	71.3 ± 0.1	492.7 ± 0.3	6.4 ± 0.6
11	52	4.5	2.5	70	69.9 ± 0.3	59.4 ± 0.2	433.6 ± 7.6	10.6 ± 1.3
12	60	4.5	2.5	70	70.0 ± 1.4	58.8 ± 0.2	453.4 ± 2.0	10.2 ± 1.4
13	52	1.5	5	70	58.4 ± 0.2	66.0 ± 0.2	457.6 ± 5.0	10.1 ± 1.4
14	60	1.5	5	70	57.9 ± 0.1	67.6 ± 0.3	474.6 ± 0.8	7.4 ± 1.1
15	52	4.5	5	70	61.3 ± 1.0	52.3 ± 0.7	417.9 ± 0.7	13.9 ± 2.1
16	60	4.5	5	70	67.4 ± 0.3	58.4 ± 0.4	441.9 ± 4.2	12.2 ± 0.4
17	50	3	3.75	60	67.3 ± 0.2	61.8 ± 0.3	426.8 ± 7.0	10.7 ± 1.0
18	62	3	3.75	60	76.4 ± 0.1	70.8 ± 0.1	536.2 ± 6.1	4.2 ± 0.6
19	56	0.75	3.75	60	36.6 ± 0.2	58.4 ± 0.2	343.8 ± 0.6	17.1 ± 0.4
20	56	5.25	3.75	60	66.1 ± 0.4	56.7 ± 0.1	417.1 ± 1.8	10.3 ± 1.8
21	56	3	1.875	60	76.3 ± 0.2	74.5 ± 0.2	535.5 ± 1.8	3.1 ± 0.4
22	56	3	5.625	60	67.2 ± 1.0	64.9 ± 0.2	478.0 ± 1.2	6.2 ± 1.2
23	56	3	3.75	45	62.7 ± 0.5	67.8 ± 0.2	494.5 ± 3.0	5.1 ± 0.7
24	56	3	3.75	75	72.0 ± 0.5	64.2 ± 0.1	478.5 ± 0.6	7.4 ± 1.4
25	56	3	3.75	60	73.7 ± 0.3	68.9 ± 0.3	500.8 ± 2.0	4.4 ± 0.9
26	56	3	3.75	60	72.4 ± 0.5	67.6 ± 0.3	488.5 ± 1.7	4.8 ± 0.5
27	56	3	3.75	60	72.8 ± 0.5	67.9 ± 0.2	504.0 ± 1.1	4.9 ± 0.9

**Table 5:** Analysis of variance of the response surface reduced quadratic models for fermented dough height and height, volume, and firmness of bread prepared with ‘O-free’ flour.

Response	Source	DF	Sum of Squares	Mean Square	F Value	R^2^
Fermented dough height	Model	8	2690.54	336.32	59.05 ***	0.963
	Residue	18	102.51	5.70		
	Total	26	2793.05			
Bread height	Model	6	577.46	96.24	18.76 ***	0.849
	Residue	20	102.60	5.13		
	Total	26	680.06			
Bread volume	Model	6	47,689.33	7948.22	15.79 ***	0.826
	Residue	20	10,064.36	503.22		
	Total	26	57,753.69			
Bread firmness	Model	8	301.15	37.64	9.42 ***	0.807
	Residue	18	71.92	4.00		
	Total	26	373.06			

*** indicated significance at *p* < 0.0001.

**Table 6:** Comparison of predicted, experimental actual, and center point values for confirming the optimized solution.

Factors	Confirmation Test		Center Point
	Predicted Value	Experimental Value	
Water amount (g)	60	60	56
Yeast amount (g)	3.1	3.1	3
Mixing time (min)	2.5	2.5	3.75
Fermentation time (min)	54.2	54.2	60
Bread height (mm)	71.2	78.7	67.9
Bread volume (mL)	522.9	562.5	504
Bread firmness (N)	3.3	3.7	4.9

## Data Availability

The data presented in this study are available upon request from the corresponding author.
